# Supplementary material for: Promoting Affirmative Transgender Health Care Practice Within Hospitals: An IPE Standardized Patient Simulation for Graduate Health Care Learners
Source: MedEdPORTAL. 2019 Dec 13;15:10861. doi: 10.15766/mep_2374-8265.10861 (PMC7010321; doi:10.15766/mep_2374-8265.10861)
Supplement: Supplementary file 1 — A. Logistical Requirements.docx B. Facilitator Guide.docx C. Standardized Patient Case Development Tool.docx D. IP Core Competencies Critique for ED Video.docx E. IP Behaviors for Team Huddle and Discharge Planning.docx F. ED Video.mp4 G. Guidelines for Student and Facilitator Debriefs.docx H. Posttest Assessment Survey.pdf [file mep-15-10861-s001.zip › _Educational_Summary_Report_10861.pdf]

MedEdPORTAL® | The AAMC Journal of Teaching and Learning Resources

Original Publication
Open Access

Promoting Affirmative Transgender Health Care Practice Within Hospitals: An IPE Standardized Patient Simulation for Graduate Health Care Learners

Emily L. McCave, PhD, MSW*, Dennis Aptaker, OTD, MOT, Kimberly D. Hartmann, PhD, MHS, Rebecca Zucconi, MD

*Corresponding author: emily.mccave@qu.edu

Abstract

Introduction: Transgender patients frequently experience discrimination within health care settings due to provider lack of knowledge and bias resulting in poor service delivery. Team-based interprofessional collaboration is becoming a best practice for health professionals to improve patient-centered care and address these health disparities. **Methods:** A team-based interprofessional education simulation activity was developed as a teaching activity at a university for graduate health care learners in medicine, nursing, occupational therapy, physical therapy, physician assistant, social work, and health care administration programs over 2 years ($N = 494$). The simulation focused on a transgender patient brought to the emergency department (ED) after a workplace assault. Students were placed in interprofessional teams and asked to critique the initial ED interaction with the patient and then complete a team huddle and discharge planning meeting with a standardized patient. Student preparedness to engage in the Interprofessional Education Collaborative (IPEC) competencies was assessed through a posttest measure. **Results:** Student learners reacted overwhelmingly positively to the activities of the workshop. The averaging of 2 years of data yielded students responses of strongly agree and agree at 90% or higher for all IPEC core competencies, as well as for educational objectives of the workshop. **Discussion:** Reducing the structural, interpersonal, and individual stigma experienced by transgender patients requires institutions to offer experiential learning opportunities for future health care providers. This interprofessional education simulation experience focusing on transgender patients calls attention to the negative impact of stigma while also promoting competency in interprofessional practice.

Keywords
Social Work, Affirmative Practice, Transgender Patient, Communication Skills, Cultural Competence, Gender Identity, Diversity, Inclusion, Health Equity, Human Sexuality, Interprofessional Education, LGBTQ+, Sexual and Gender Minorities, Emergency Medicine, Standardized Patient

Educational Objectives

By the end of this activity, learners will be able to:

1. Describe the unique and overlapping professional roles and responsibilities of the providers on a health care team.
2. Communicate effectively as an interprofessional health care team during a team huddle and a discharge planning simulation for a transgender patient.
3. Develop shared ethics as an interprofessional team during a simulation experience.
4. Critique interprofessional teamwork during a team huddle and simulated discharge planning meeting.
5. Apply affirmative practice skills with a transgender patient during a simulated discharge planning meeting.

Citation:
McCave EL, Aptaker D, Hartmann KD, Zucconi R. Promoting affirmative transgender health care practice within hospitals: an IPE standardized patient simulation for graduate health care learners. *MedEdPORTAL.* 2019;15:10861.
https://doi.org/10.15766/mep_2374-8265.10861

Introduction

Transgender patients disproportionately experience discrimination within the health care system.[1-3] In 2015, the National Center for Transgender Equality conducted the largest national survey of transgender people, with more than 27,000 surveys completed from all 50 states and territories.[4] In the domain of health care, the executive summary reported that within the past year, one-third of respondents had experienced a negative encounter with a health provider, and almost one-quarter had avoided seeking medical care due to fear of being mistreated as a transgender person. Barriers to accessing health care range from failure or refusal to provide appropriate and sensitive medical care to engaging in harassment or violent

Copyright © 2019 McCave et al. This is an open-access publication distributed under the terms of the Creative Commons Attribution license.

behavior toward transgender patients. As a consequence, both preventative care and treatment for illness and injury are often avoided by transgender patients. Discriminatory behaviors often emanate from the health care team's existing attitudes and lack of knowledge and skills in providing affirming care to transgender patients.[4] Health promotion of transgender individuals has been recognized at the national policy level as well. One of the goals of the Heathy People 2020 initiative out of the US Department of Health and Human Services is to improve the health, safety, and well-being of transgender individuals.[5] To achieve that goal, the structural, interpersonal, and individual stigma experienced by transgender individuals needs to be reduced.[6] All three levels of stigma contribute to the health disparities of transgender individuals through mechanisms such as lack of provider training and education (i.e., structural stigma), discriminatory behaviors by health care providers (i.e., interpersonal stigma), and personal avoidance of health care systems and providers (i.e., individual stigma). One of the ways to intervene at the structural and interpersonal levels is to expose health care learners to transgender health content and provide opportunities for future health care providers to observe and engage in health care practice situations with transgender patients.[6]

There is limited literature available on the preparation of health care learners to address the levels of stigma impacting transgender patients. What is available is focused on preparing medical students uniprofessionally to conduct interviews and examinations with transgender patients using standardized patients or educational films.[7-9] Additionally, there is limited literature on interprofessional education (IPE) activities for health care learners to promote affirmative practice with transgender patients. The literature thus far has centered on health equity and uses case studies for discussion but does not highlight the use of standardized patients.[10-12] There are multiple benefits of choosing standardized patients as a pedagogical tool, such as providing exposure to clinical scenarios that may not be experienced by all learners in clinical rotations, as well as offering a consistent patient experience that allows for immediate feedback within a supportive environment; this can increase confidence and decrease anxiety, which may reduce apprehension during future patient encounters.[13,14] When considering this particular marginalized population, utilizing standardized patients also provides an experience in which learners can practice their skills without risking further stigmatization of an actual patient.

Utilizing standardized patients within the context of an interprofessional teaching activity represents an untapped resource for providing health care learners with the critical education and skills development needed to address these health disparities. With team-based interprofessional collaboration becoming a best practice in health care education programs, there is added benefit to creating teaching activities that address a variety of health disciplines rather than targeting only one profession.[15] The health disciplines targeted for this teaching activity include medicine, nursing, occupational therapy, physician assistant, physical therapy, and social work programs. The core competencies for interprofessional collaborative practices, created by the Interprofessional Education Collaborative (IPEC) to prepare future health professionals for enhanced team-based care of patients and improve population health outcomes, serve as the foundation of this teaching activity.[16] All four core competencies—focused shared values and ethics, roles and responsibilities, communication, and teams and teamwork—are targeted in this teaching activity centered on treating a transgender patient in a hospital setting.

Methods

Workshop Development

This IPE simulation for graduate health care learners was created by an interprofessional team of university faculty from the Schools of Medicine, Nursing, and Health Sciences (i.e., physician assistant, social work, occupational therapy, and physical therapy programs). All materials, both written documents and the emergency department (ED) video, were created by the authors. To ensure the authenticity of the case and avoid stereotyping, two transgender individuals were consulted during the case development and gave feedback on the drafted case. One of several signature seminars sponsored by the university's Center for Interprofessional Healthcare Education (CIHE), the workshop aimed to increase interprofessional practice knowledge and skills when working as a member of a health care team with a transgender patient. Student learners were expected to have foundational knowledge in patient assessment, intervention, and discharge planning in their respective disciplines. Members on the planning committee considered where the learning activity would best fit within their respective programs' curricula at both the graduate and undergraduate levels. For all programs represented, graduate learners were chosen. For medicine, physician assistant, occupational therapy, and social work programs, graduate learners in their first year were selected. The doctor of nursing program and doctor of physical therapy program did not specify a target year for their graduate learners. Facilitators needed prerequisite knowledge of the four core IPEC competencies.

Copyright © 2019 McCave et al. This is an open-access publication distributed under the terms of the Creative Commons Attribution license.

Preworkshop Preparation

Coordinator: A faculty member from the planning committee volunteered to serve as the coordinator and oversaw the logistical aspects of the workshop with support from the planning team and the director of the CIHE. In creating and implementing the simulation workshop, logistical requirements regarding the personnel, equipment/supplies, space, and time resources were identified (Appendix A). Seminar rooms within the university served as a conference room within a hospital. The coordinator recruited facilitators, comprising both faculty of varying health disciplines and graduate students who had participated in the workshop the prior year, had greater than 80 hours of IPE, or had established expertise in working with transgender individuals. All registered student learners were assigned to interprofessional teams to maximize the representation from the disciplines participating. Two facilitators from different disciplines were assigned to each student team. If there was not representation of a specific discipline among a student team, a facilitator from that discipline was assigned to that student team.

Standardized patients: The coordinator worked directly with the director of the Standardized Patient and Assessment Center (SPAC) to arrange the selection and training of the standardized patients. The coordinator provided training to the standardized patients in which they watched the ED video (Appendix F), reviewed the standardized patient case (Appendix C), and participated in a question-and-answer period. The coordinator shared anecdotes of health care experiences of transgender individuals, particularly those involving misgendering and the challenges faced when trying to access routine, specialized, and emergency health care, to highlight the stigmatization that occurs and how it can negatively impact the overall well-being of transgender individuals. As the SPAC did not have any available standardized patients who were transgender men, cisgender females were recruited as standardized patients to portray a transgender man with masculine gender expression. This was done in consultation with two members of the transgender community, with the recognition that misgendering most often happens in the early stages of transition, when visual cues lead to inaccurate assumptions during social interactions. Given varying ages of available standardized patients, no specific age was required beyond being 18 years or older, although target standardized patients were in their 20s or 30s. A standardized patient was assigned to each interprofessional team.

Facilitators: The coordinator provided training for facilitators in preparation for the event. Facilitators were each given a folder with workshop materials, including the facilitator guide (Appendix B), the standardized patient case (Appendix C), and two student handouts (Appendices D and E). Facilitators were shown the ED video simulation (Appendix F) and presented with each aspect of the workshop, using the facilitator guide as the primary reference document. Facilitators were emailed a preworkshop reading packet prior to the workshop. This reading packet contained the IPEC core competencies,[16] a description of the roles and responsibilities of typical health care providers on a health care team, a description of the rights afforded to transgender individuals related to bathroom access by the Occupational Safety and Health Administration,[17] a guide to workers' compensation in the state, and a description of best practices and terminology affirming to transgender patients.[18,19]

Student learners: Prior to the workshop, student learners were emailed the same preworkshop reading packet as the facilitators, as noted above.

Workshop Components

Stonewall Speakers: Following opening remarks from the coordinator, student learners and faculty attended a panel presentation from a local community organization—Stonewall Speakers. As part of Connecticut's Stonewall Foundation, Stonewall Speakers provides educational outreach opportunities promoting awareness and understanding for lesbian, gay, bisexual, and transgender (LGBT) people. The speaking engagement consisted of two speakers who shared their personal stories as health care consumers, as well as a question-and-answer opportunity for student learners and faculty. The Stonewall Speakers' contributions lasted approximately 40 minutes and helped to keep the human experience central throughout the simulation and interprofessional event.

Simulation: Students began the simulation portion of the workshop by watching the 10-minute ED video simulation (Appendix F) that had been recorded in the SPAC hospital examination room featuring a standardized patient (Peter Jacobs), a physician, a nurse, a social worker, and a medical assistant. The simulation showed Peter in the ED after a workplace assault resulting in an ankle injury. Once the video concluded, the facilitators took their student teams to their respective breakout rooms. The facilitators led a 15-minute discussion of how the interprofessional core competencies and standards for affirmative practice were upheld and violated by the behaviors of the providers seen in the video (Appendix D). Students then completed a 10-minute team huddle to prepare for the initial discharge planning meeting with Peter. The team discussed what information was still needed, which providers might be needed

Copyright © 2019 McCave et al. This is an open-access publication distributed under the terms of the Creative Commons Attribution license.

for discharge planning, ideas for potential referrals for aftercare needs, and important aspects related to transgender affirmative practice. The facilitators offered ideas to the student team if necessary and led a 15-minute discussion on their teamwork during the huddle. The standardized patient (Peter) was then brought into each room; the student team conducted a 25-minute discharge planning meeting with Peter. The team was tasked with focusing on interprofessional practice behaviors and affirmative practice while putting Peter at the center of the team (Appendix E). Following this, the standardized patients gave 5 minutes of positive and constructive feedback to the student teams on their patient experience as Peter. Facilitators then led a 10-minute team debrief on the discharge planning experience as it related to interprofessional practice behaviors and affirmative practice.

Large-group debriefing and assessment: Following the conclusion of the simulation, the students participated in a 20-minute large-group debrief (Appendix G) and were sent a postevent electronic survey (Appendix H). Once student learners were dismissed, facilitators participated in their own debrief and then were sent a postevent electronic survey. The student and facilitator surveys collected demographic data, satisfaction data, and ability to practice the IPEC subcompetencies that had been selected as outcomes for the event. The survey questions related to the IPEC subcompetencies were adapted from Alan Dow's 2012 IPEC Competency Survey, which put a Likert scale to the IPEC subcompetencies.[16,20]

Results

This simulation involved students from a range of health disciplines and professions. A breakdown of graduate health care learners by profession is outlined in Table 1. On average, each occurrence of the event involved approximately 18 standardized patients, 28 faculty facilitators, and six student facilitators. Over 2 years, 494 students participated in the workshop (Table 2); the participation of student learners doubled in size the second year. The facilitators represented several programs at the university, including medicine, nursing, occupational therapy, physical therapy, physician assistant, and social work.

Table 1. Student Learner Breakdown

Discipline	2017 No. (%)	2018 No. (%)	Total No. (%)
Occupational therapy	79 (49)	85 (26)	164 (33)
Physician assistant	55 (34)	53 (16)	108 (22)
Medicine	2 (1)	81 (24)	83 (17)
Physical therapy	12 (7)	53 (16)	65 (13)
Social work	5 (3)	33 (10)	38 (8)
Nursing	8 (5)	28 (8)	36 (7)
Total	161 (100)	333 (100)	494 (100)

Table 2. Survey Response Rates

Discipline	2017 No. (%)	2018 No. (%)	Total No. (%)
Occupational therapy	61 (48)	39 (26)	100 (49)
Medicine	2 (2)	32 (21)	34 (7)
Physical therapy	11 (9)	28 (19)	39 (8)
Physician assistant	48 (37)	27 (18)	75 (15)
Nursing	0 (0)	12 (8)	12 (2)
Social work	5 (4)	12 (8)	17 (3)
Business administration	1 (1)	0 (0)	1 (0)
Total responses	128 (79)	150 (45)	278 (56)
Total participants	161 (100)	333 (100)	494 (100)

Students completed an electronic postevent survey regarding their experiential learning from the workshop. Data were gathered on demographic characteristics of the students, their learning connected to the IPEC competencies, and feedback on all major aspects on the workshop. On average, 95% of the students were required to attend the workshop for a course they were enrolled in during the spring semester. Aside from the nursing students in 2017 and business administration students for 2017 and 2018, all remaining disciplines participated in the survey with similar rates of response as their respective participation levels in the workshop.

The postevent survey was timed to be distributed electronically during the large student debrief to encourage participation. A Likert scale was utilized to assess the value of each component of the event (Table 3). A majority (93%) of student responses identified the addition of transgender individuals sharing their personal experiences as useful or very useful. Participants valued the simulation video (62%) and small team debriefing of their observations (68%) as useful or very useful. Responders identified increased value during the team huddle and use of the observation checklist (82%), as well as the discharge planning meeting with the standardized patient (85%), over the past 2 years of the event.

Table 3. Workshop Value by Component[a]

Component	2017[b] No. (%)	2018[c] No. (%)	Total[d] No. (%)
Stonewall Speakers	120 (93)	141 (94)	261 (93)
Discharge planning meeting with standardized patient and observation checklist	117 (91)	121 (80)	238 (85)
Team huddle and observation checklist	109 (85)	119 (79)	228 (82)
Team debrief on video simulation	100 (78)	87 (58)	187 (67)
Video simulation	92 (71)	80 (53)	172 (61)
Large student debrief	78 (60)	61 (40)	139 (50)

[a]Rated useful or very useful on a 5-point scale (1 = *not useful*, 2 = *somewhat useful*, 3 = *neutral*, 4 = *useful*, 5 = *very useful*).
[b]n = 128.
[c]n = 150.
[d]n = 278.

Copyright © 2019 McCave et al. This is an open-access publication distributed under the terms of the Creative Commons Attribution license.

After completing the demographic and component value scales, students were also asked to rate their level of preparation utilizing IPEC competencies as a result of the workshop. This was accomplished by asking to what extent participants felt better prepared to engage in interprofessional collaborative practices. Table 4 illustrates participants' preparation implementing IPEC core competencies following participation in the workshop.[16,18] Student learners reacted overwhelmingly positively to the activities of the workshop. The averaging of 2 years of data yielded student responses of strongly agree or agree at 90% or higher for all IPEC core competencies, as well as for the educational objectives of the workshop. Students identified their preparation to maintain values, ethics, and affirming practice when working on an interprofessional team to discharge a transgender patient at 93%. Furthermore, students identified their preparation for teamwork (90%), effective communication (90%), and collaboration with a variety of health care professions (91%) to effectively meet the needs of a transgender patient on discharge from the hospital setting during this simulation. Overall, student learners rated themselves as being prepared to engage in behaviors consistent with the core competencies for interprofessional practice. This was echoed during the facilitator and student debriefs when several examples were given of student learners having demonstrated interprofessional practice behaviors during the team huddle and discharge planning meeting that were in line with the core competencies. That said, facilitators did note that some students were much more active in their participation than others.

Discussion

Implications

The results shown in Table 3 indicate that the active dialogue and standardized patient learning sessions (e.g., community presenters who were transgender, team debrief, team huddle, and discharge planning) were viewed as most useful. The video simulation and the large debrief yielded lower-value scores

Table 4. IPEC Competency Postevent Survey Results[a]

IPEC Competency	After the IPE Workshop, I Feel Better Prepared to:	2017[b] No. (%)	2018[c] No. (%)	Total[d] No. (%)
Values and ethics				
	Place interests of patients and populations at center of interprofessional health care delivery and population health programs and policies, with the goal of promoting health and health equity across the life span.	119 (93)	134 (89)	253 (91)
	Respect the dignity and privacy of patients while maintaining confidentiality in the delivery of team-based care.	121 (97)	134 (89)	255 (91)
	Embrace the cultural diversity and individual differences that characterize patients, populations, and the health team.	121 (97)	135 (90)	256 (92)
	Respect the unique cultures, values, roles/responsibilities, and expertise of other health professions and the impact these factors can have on health outcomes.	122 (95)	137 (91)	259 (93)
	Maintain competence in one's own profession appropriate to scope of practice.	119 (93)	132 (89)	251 (90)
Role and responsibilities				
	Communicate one's roles and responsibilities clearly to patients, families, community members, and other professionals.	120 (93)	131 (89)	253 (91)
	Recognize one's limitations in skills, knowledge, and abilities.	121 (94)	135 (91)	256 (92)
	Engage diverse professionals who complement one's own professional expertise, as well as associated resources, to develop strategies to meet specific health and health care needs of patients and populations.	121 (94)	130 (87)	251 (90)
	Explain the roles and responsibilities of other providers and how the team works together to provide care, promote health, and prevent disease.	114 (89)	129 (86)	243 (87)
Interprofessional communication				
	Listen actively and encourage ideas and opinions of other team members.	121 (95)	132 (89)	253 (91)
	Give timely, sensitive, instructive feedback to others about their performance on the team, responding respectfully as a team member to feedback from others.	118 (92)	122 (82)	240 (86)
	Use respectful language appropriate for a given difficult situation, crucial conversation, or conflict.	120 (94)	128 (86)	248 (89)
Teamwork				
	Engage health and other professionals in shared patient-centered and population-focused problem solving.	121 (95)	134 (90)	255 (91)
	Reflect on individual and team performance for individual, as well as team, performance improvement.	120 (93)	134 (90)	254 (91)
	Use available evidence to inform effective teamwork and team-based practices.	117 (92)	119 (80)	236 (84)

Abbreviations: IPE, interprofessional education; IPEC, Interprofessional Education Collaborative.
[a]Rated agree or strongly agree on a 5-point scale (1 = *strongly disagree*, 2 = *disagree*, 3 = *neutral*, 4 = *agree*, 5 = *strongly agree*).
[b]$n = 128$.
[c]$n = 150$.
[d]$n = 278$.

Copyright © 2019 McCave et al. This is an open-access publication distributed under the terms of the Creative Commons Attribution license.

and were the learning activities not directly associated with dialogue with transgender individuals from the community or the standardized patients. This suggests that both centering the voices of transgender individuals and providing interactive opportunities utilizing standardized patients are key when developing learning activities aimed at improving health care practice with transgender patients. The results in Table 4 demonstrate that the learners felt better prepared to engage in the IPEC core competencies as related to transgender health due to the participation in the learning activity. The self-reported data were supported by observations reported by both facilitators and student learners during the debriefs. Given this, the results do support that the educational objectives were met and that utilizing standardized patients within the context of the activities was a resource to promote the learning of IPEC core competencies within the scope of affirmative health practices. That said, limitations exist that should be considered when interpreting the data. These limitations include having less than a 50% response rate for the 2018 data and the potential social desirability bias in both the 2017 and 2018 data. An additional limitation worth noting is that the impact of the educational activity has not been measured in actual clinical settings, either immediately after the workshop or longitudinally. Finally, the conclusions from the results would be strengthened with qualitative data. An open-ended question was included in the 2017 survey; however, few participants responded, and when survey software platforms were switched, the research team lost access to the qualitative data. Due to the low response rate, the open-ended question was removed from the 2018 survey.

Value of Learning Activity

This simulation has three critical areas of value. First, this learning experience provides multimodal methods to explore the complexities of transgender health and the potential intrinsic biases or discomfort of providers while illustrating the usefulness of interprofessional practice as a strategy to reduce the stigma faced by this population. During the facilitator debrief, facilitators discussed observing discomfort in some student learners, both verbally and nonverbally, when interacting with Peter. This was also brought up during the student debrief, when students shared that they were anxious because they had never interacted with someone who was transgender and were worried that they would make a mistake. While some stated that they tried to approach the interaction as a learning opportunity and admittedly stumbled at times, others indicated that rather than speaking up, they relied on their team members to engage with Peter and tried to learn from their peers. Student learners shared that hearing the stories of transgender individuals prior to the simulation highlighted how important it is for providers to use affirming language and to consider the larger social, financial, and emotional challenges faced by transgender individuals that can impact health promotion. It seems that having the opportunity to first hear from local transgender health consumers and then practice patient interaction in a low-risk setting allowed students to wrestle with how to negotiate their lack of knowledge and skills in providing affirmative care to a transgender patient.

Second, the learning activity offers a structured opportunity for interprofessional teams of graduate health care learners to engage in the four core interprofessional practice competencies by experiencing the diverse roles and responsibilities of team members, practicing communication skills and teamwork via a team huddle and discharge planning meeting, and demonstrating shared ethics by providing affirmative health care to a transgender patient. During both the facilitator and student debriefs, it was shared that learners gained new information about the value of other professions, such as social work and occupational therapy, which possessed critical knowledge of workers' compensation and insurance coverage for gender-affirming surgery. Facilitators and student learners alike stated that the learning activity required shared leadership and open communication, particularly during the team huddle and discharge planning meeting.

Third, the learning activity has value for faculty developers and faculty facilitators in that all four of the IPEC core competencies are essential to in the development and implementation of the learning activity. This reinforced the IPEC core competencies for faculty and promoted genuine role modeling by faculty to student learners. For first-time facilitators, being able to model skills in teamwork, communication, and understanding of roles and responsibilities seemed to be an area of confidence, as discussed in the facilitator debrief. Notably, some of the repeat facilitators expressed that they felt more confident during the second year in being able to model affirmative health care practice when student learners were uncertain and in articulating how embracing affirmative practice behaviors as a team exemplified shared ethics across disciplines.

Lessons Learned

Lessons learned through the development, implementation, and evaluation are similar to lessons documented in other interprofessional learning literature but are useful to reiterate. Despite the pragmatic issues often associated with designing and implementing interprofessional learning activities, the power of learning with and from each other promotes quality dialogue

Copyright © 2019 McCave et al. This is an open-access publication distributed under the terms of the Creative Commons Attribution license.

and problem-solving opportunities for learners beyond what can be accomplished through uniprofessional education.[15,16,21,22] Developing pilot educational experiences has merit; however, scaling up in size is a significant challenge unless established as a goal and strategized in the design phase. While in the design phase, it is essential to develop a script and train the standardized patients to emphasize the focus on the complex health care needs of a transgender patient. This includes inviting members of the transgender community to share their experiences as health care patients with the standardized patients to provide much-needed context. Continuing to reflect on the learning experience and adjusting based on both learner feedback and faculty feedback are important to replicate successes. Additionally, further modifications may need to be made if unexpected opportunities arise that could enhance the learning activity. For example, if by way of other collaborative efforts a nearby school of pharmacy at another university asks to participate in the future, it would be beneficial to seek its faculty's input on the case and the facilitator guide to ensure that the pharmacy role is appropriately represented and its students are incorporated fully into the learning activity.

A significant lesson was incorporating into the learning experience the perspectives of transgender individuals as health care consumers from the community. The results support this, with the learners (98% in 2017 and 93% in 2018) indicating that the transgender panel from the community was valued as very useful or useful. For replication purposes, it is recommended that institutions conduct outreach to local and/or state LGBT consumer-focused organizations when there are existing partnerships; the Stonewall Speakers had served as speakers for other academic programs within the university and so were already known to those involved with this learning activity. If there are no existing partnerships, a useful place to start is by researching whether there is a local LGBT speakers bureau. If that is not an option, it may be beneficial to contact local organizations that serve LGBT individuals to request possible ideas or ask those involved with the learning activity to consider their own social networks.

In regard to utilizing standardized patients, it is critical that institutions are intentional about who they are recruiting for this learning activity. Ideally, transgender men would be recruited for the role of Peter so as to provide the most accurate and authentic representation. That said, institutions may find that they have limited representation of individuals who identify as transgender men within a standardized patient pool. This may signal an opportunity to reexamine current outreach and recruitment strategies. Initially, institutions may need to utilize as least some actors who are cisgender and thus playing a role about which they may have limited understanding. In such circumstances, recruitment of standardized patients should be highly selective, with training built in to highlight the complex social, emotional, financial, and medical aspects of the case. Part of the training should include shared experiences from those within the transgender community to increase empathy and authentic representation. Depending on an institution's unique environmental context, standardized patient recruitment could include cisgender men or women with consultation from members of the transgender community. In connection with this, the length of time that Peter has been taking testosterone could be modified to fit the available standardized patient pool given the hormone's effect on secondary sex characteristics such as facial hair and voice (e.g., less than 1 month on testosterone if utilizing cisgender females or several months on testosterone if utilizing cisgender men). Given that not all institutions have access to standardized patients, careful consideration needs to be given to how best to proceed with recruiting and training individuals to take on the role of Peter, including consultation with members from the transgender community.

Some potential obstacles to replicating this simulation include the resources available within the institution to develop and implement the workshop. Such resources include the time and commitment by facilitators, the space required to implement the various components of the workshop, the availability of varied graduate health learners to participate, and the availability of standardized patients. In considering facilitator resources, having two interprofessional facilitators for each learner team requires a large pool from which to draw; smaller institutions may want to consider drawing from part-time faculty, clinical preceptors, or students with strong facilitation skills and/or knowledge of the IPEC competencies. As a result, institutions are encouraged to start with a smaller-scale workshop to establish a pool of standardized patients and facilitators. Based on this method, the authors' university has been able to maintain a stable core group of facilitators and standardized patients who are excited to participate annually and recruit others as needed. Given that many disciplines assign this workshop as a course requirement, it may be necessary to offer the simulation multiple times in a year or to rotate availability to disciplines.

It is important to note that the simulation focuses on the IPEC competencies rather than content learning on the health care diagnosis and interventions for this case. In the authors' experience, facilitators and student learners may get stuck on

Copyright © 2019 McCave et al. This is an open-access publication distributed under the terms of the Creative Commons Attribution license.

technical aspects of the case (e.g., Peter's insulin dosage). A consideration for data collection is to ensure alignment between the learning objectives and the assessment measures. To that point, student learners may experience assessment fatigue if attending multiple interprofessional events; having a limited number of learning objectives and assessment questions is recommended. For data analysis, it is worthwhile to project needed resources as the workshop grows in size. Many online survey software providers offer free subscriptions for data collection and analysis up to a certain number of respondents. This university identified the need to purchase a paid subscription for survey collection and analysis software to accommodate the large number of participants.

Future Directions

Future directions include refinement of the workshop to advance those components the graduate health care learners identified as most valuable. Given the evaluation feedback on the personal and community perspective, the developers of this workshop are planning to increase the diversity of individual narratives to represent a broader perspective from transgender individuals receiving health care services. Additionally, a future research goal is to collect longitudinal data by following students who have attended this workshop into their future clinical rotations or as alumni. The aim would be to conduct a focus group to determine if and/or how the workshop has impacted participants' current clinical practice or to have clinical supervisors view and rate an interaction with either an actual transgender patient or a simulated patient. Another possible avenue being considered is to conduct the workshop at a clinical site where students are already on interprofessional teams serving alongside health care professionals. Both learners and credentialed providers could likely benefit from the workshop, with an opportunity for both immediate and longitudinal data collection. There have also been early discussions within the planning committee to explore ways to pair this event with other LGBTQ-focused events during times of national recognition, such as PRIDE Month, LGBT History Month, or Transgender Awareness Month. Such an activity may include a book club or an event focused on policy advocacy. In doing so, this workshop could serve as one critical learning opportunity in a larger array of events aimed at reducing stigma and providing students with an opportunity to enhance their comfort and competence in meeting the needs of this marginalized yet resilient population. Such exploration could lead to new collaborations with interested stakeholders both within the university community and beyond. Finally, this event establishes an opportunity for a larger discussion on how to infuse content on the health care needs of transgender individuals into discipline-specific curricula. Although reducing the individual, interpersonal, and structural stigma faced by this population requires more than a single learning activity, providing graduate health care learners with an opportunity to practice delivering affirmative practice with standardized patients in the context of an interprofessional team is an important step toward achieving that aim.

Appendices

A. Logistical Requirements.docx
B. Facilitator Guide.docx
C. Standardized Patient Case Development Tool.docx
D. IP Core Competencies Critique for ED Video.docx
E. IP Behaviors for Team Huddle and Discharge Planning.docx
F. ED Video.mp4
G. Guidelines for Student and Facilitator Debriefs.docx
H. Posttest Assessment Survey.pdf

All appendices are peer reviewed as integral parts of the Original Publication.

Emily L. McCave, PhD, MSW: Associate Professor, Department of Social Work, Quinnipiac University; Assistant Director, Center for Interprofessional Healthcare Education, Quinnipiac University

Dennis Aptaker, OTD, MOT: Clinical Assistant Professor, Department of Occupational Therapy, Quinnipiac University

Kimberly D. Hartmann, PhD, MHS: Professor, Department of Occupational Therapy, Quinnipiac University; Director, Center for Interprofessional Healthcare Education, Quinnipiac University

Rebecca Zucconi, MD: Assistant Professor, Department of Medical Sciences, Frank H. Netter MD School of Medicine at Quinnipiac University

Acknowledgments
The authors would like to thank Dr. Aaron Bernard and Gabbriel Ceccolini for their assistance in developing the standardized patient case, along with the faculty, staff, and students who served as actors for the videos and all facilitators and standardized patients who have made this activity possible. Thanks to Nicholas Donohue for assisting with the final editing of the manuscript.

Disclosures
None to report.

Funding/Support
None to report.

Copyright © 2019 McCave et al. This is an open-access publication distributed under the terms of the Creative Commons Attribution license.

Prior Presentations
McCave EL, Hartmann KD, Aptaker D, Zucconi R. Promoting affirmative transgender health care practice within hospitals: an IPE standardized patient simulation. Workshop presented at: 64th Council on Social Work Education Annual Program Meeting; November 10, 2018; Orlando, FL.

Ethical Approval
Reported as not applicable.

References

1. Cicero EC, Black BP. "I was a spectacle . . . a freak show at the circus": a transgender person's ED experience and implications for nursing practice. *J Emerg Nurs*. 2016;42(1):25-30. https://doi.org/10.1016/j.jen.2015.08.012
2. Grant JM, Mottet LA, Tanis J, Herman JL, Harrison J, Keisling M. National Transgender Discrimination Survey Report on Health and Health Care: findings of a study by the National Center for Transgender Equality and the National Gay and Lesbian Task Force. National LGBT Cancer Network website. https://cancer-network.org/wp-content/uploads/2017/02/National_Transgender_Discrimination_Survey_Report_on_health_and_health_care.pdf. Published October 2010. Accessed January 25, 2018.
3. Redfern JS, Sinclair B. Improving health care encounters and communication with transgender patients. *J Commun Healthc*. 2014;7(1):25-40. https://doi.org/10.1179/1753807614Y.0000000045
4. Executive summary. In: James SE, Herman JL, Rankin S, Keisling M, Mottet L, Anafi M. *The Report of the 2015 U.S. Transgender Survey*. Washington, DC: National Center for Transgender Equality; 2016:3-17.
5. Lesbian, gay, bisexual, and transgender health. Office of Disease Prevention and Health Promotion's Healthy People 2020 website. https://www.healthypeople.gov/2020/topics-objectives/topic/lesbian-gay-bisexual-and-transgender-health. Updated January 24, 2019. Accessed January 25, 2019.
6. White Hughto JM, Reisner SL, Pachankis JE. Transgender stigma and health: a critical review of stigma determinants, mechanisms, and interventions. *Soc Sci Med*. 2015;147:222-231. https://doi.org/10.1016/j.socscimed.2015.11.010
7. Ahmad T, Yan H, Guidolin K, Foster P. Medical queeries: transgender health care. *MedEdPORTAL*. 2015;11:10068. https://doi.org/10.15766/mep_2374-8265.10068
8. Greene RE, Hanley K, Cook TE, Gillespie C, Zabar S. Meeting the primary care needs of transgender patients through simulation. *J Grad Med Educ*. 2017;9(3):380-381. https://doi.org/10.4300/JGME-D-16-00770.1
9. Underman K, Giffort D, Hyderi A, Hirshfield LE. Transgender health: a standardized patient case for advanced clerkship students. *MedEdPORTAL*. 2016;12:10518. https://doi.org/10.15766/mep_2374-8265.10518
10. Braun HM, Ramirez D, Zahner GJ, Gillis-Buck EM, Sheriff H, Ferrone M. The LGBTQI health forum: an innovative interprofessional initiative to support curriculum reform. *Med Educ Online*. 2017;22(1):1306419. https://doi.org/10.1080/10872981.2017.1306419
11. Calzo JP, Melchiono M, Richmond TK, et al. Lesbian, gay, bisexual, and transgender adolescent health: an interprofessional case discussion. *MedEdPORTAL*. 2017;13:10615. https://doi.org/10.15766/mep_2374-8265.10615
12. Leslie KF, Steinbock S, Simpson R, Jones VF, Sawning S. Interprofessional LGBT health equity education for early learners. *MedEdPORTAL*. 2017;13:10551. https://doi.org/10.15766/mep_2374-8265.10551
13. Panzarella K, Rivers L, Bright B, et al. Using actors as simulated patients for interprofessional education. *Med Sci Educ*. 2013;23(suppl 3):438-448. https://doi.org/10.1007/BF03341666
14. Beigzadeh A, Bahmanbijari B, Sharifpoor E, Rahimi M. Standardized patients versus simulated patients in medical education: are they the same or different. *J Emerg Pract Trauma*. 2016;2(1):25-28. https://doi.org/10.15171/jept.2015.05
15. Khalili H, Orchard C, Laschinger HKS, Farah R. An interprofessional socialization framework for developing an interprofessional identity among health professions students. *J Interprof Care*. 2013;27(6):448-453. https://doi.org/10.3109/13561820.2013.804042
16. Interprofessional Education Collaborative. *Core Competencies for Interprofessional Collaborative Practice: 2016 Update*. Washington, DC: Interprofessional Education Collaborative; 2016.
17. Best practices: a guide to restroom access for transgender workers. Occupational Safety and Health Administration website. https://www.osha.gov/Publications/OSHA3795.pdf. Published 2015. Accessed January 23, 2019.
18. National LGBT Health Education Center. Ten things: creating inclusive health care environments for LGBT people. National LGBT Health Education Center website. https://www.lgbthealtheducation.org/wp-content/uploads/Ten-Things-Brief-Final-WEB.pdf. Published July 2015. Accessed January 23, 2019.
19. National LGBT Health Education Center. Affirmative care for transgender and gender non-conforming people: best practices for front-line health care staff. National LGBT Health Education Center website. https://www.lgbthealtheducation.org/wp-content/uploads/2016/12/Affirmative-Care-for-Transgender-and-Gender-Non-conforming-People-Best-Practices-for-Front-line-Health-Care-Staff.pdf. Updated 2016. Accessed January 23, 2019.

Copyright © 2019 McCave et al. This is an open-access publication distributed under the terms of the Creative Commons Attribution license.

20. Lockeman KS, Dow AW, DiazGranados D, et al. Refinement of the IPEC Competency Self-Assessment survey: results from a multi-institutional study. *J Interprof Care*. 2016;30(6):726-731. https://doi.org/10.1080/13561820.2016.1220928

21. Kruse J. Overcoming barriers to interprofessional education: the example of the joint position statement of the Physician Assistant Education Association and the Society of Teachers of Family Medicine. *Fam Med*. 2012;44(8):586-588.

22. Grant RW, Finnocchio LJ; California Primary Care Consortium Subcommittee on Interdisciplinary Collaboration. *Interdisciplinary Collaborative Teams in Primary Care: A Model Curriculum and Resource Guide*. San Francisco, CA: Pew Health Professions Commission; 1995.

Received: March 6, 2019
Accepted: July 26, 2019
Published: December 13, 2019

Copyright © 2019 McCave et al. This is an open-access publication distributed under the terms of the Creative Commons Attribution license.
