# Supplementary material for: Promoting Affirmative Transgender Health Care Practice Within Hospitals: An IPE Standardized Patient Simulation for Graduate Health Care Learners
Source: MedEdPORTAL. 2019 Dec 13;15:10861. doi: 10.15766/mep_2374-8265.10861 (PMC7010321; doi:10.15766/mep_2374-8265.10861)
Supplement: Supplementary file 1 — A. Logistical Requirements.docx B. Facilitator Guide.docx C. Standardized Patient Case Development Tool.docx D. IP Core Competencies Critique for ED Video.docx E. IP Behaviors for Team Huddle and Discharge Planning.docx F. ED Video.mp4 G. Guidelines for Student and Facilitator Debriefs.docx H. Posttest Assessment Survey.pdf [file mep-15-10861-s001.zip › A. Logistical Requirements.docx]

Appendix A

**Logistical Information – Personnel, Equipment/Supplies, Space, and Time Requirements**

**Personnel**

- 20 Hired standardized patients (1 SP for every group of 8-12 students)
- 40 Facilitators (Volunteer Faculty and Students)
- Volunteer Faculty Coordinator
- 4-6 Faculty members representative of disciplines attending for planning committee
- Graduate assistant

**Equipment/Supplies**

- 20 Sets of crutches or 20 wheelchairs
- 20 Ace bandages to wrap ankle
- 40 Folders for facilitators with copies of facilitator guide
- 20 Pieces of cardstock for team number placards for organizing the auditorium seating by team
- 175 Self-adhesive name badges (student name, discipline, team number, and team room number printed on it)
- 2-3 Rectangular tables for student check in
- Signage to guide students to check in and break out rooms
- Audio/visual for introductory remarks and viewing video
- 80 copies of student handouts for facilitators and break out rooms
- Electronic survey software (e.g., Survey Monkey or Qualtrics)

**Space**

- 20 seminar rooms that can hold 10-15 people (3 seats available for 2 facilitators and one SP)
- 1 large auditorium or meeting room that can hold up to 200 people
- 1 lobby area for student check in
- Training room for SPs and facilitators with audio/visual capabilities to show video

**Time Requirements**

- Faculty planning committee – 4 hours a month at Bi-monthly meetings leading up to the event; 10 hours following event for data analysis and write up
- Faculty coordinator – 2 hours a week in the month leading up to event; the day before the event 3-4 hours for final preparations; day of event 2-3 hours before event begins and 1 hour for clean up after the event
- Graduate assistant – 5 hours a week, two weeks prior to the event and 5 hours the following week for data management
