# Supplementary material for: Promoting Affirmative Transgender Health Care Practice Within Hospitals: An IPE Standardized Patient Simulation for Graduate Health Care Learners
Source: MedEdPORTAL. 2019 Dec 13;15:10861. doi: 10.15766/mep_2374-8265.10861 (PMC7010321; doi:10.15766/mep_2374-8265.10861)
Supplement: Supplementary file 1 — A. Logistical Requirements.docx B. Facilitator Guide.docx C. Standardized Patient Case Development Tool.docx D. IP Core Competencies Critique for ED Video.docx E. IP Behaviors for Team Huddle and Discharge Planning.docx F. ED Video.mp4 G. Guidelines for Student and Facilitator Debriefs.docx H. Posttest Assessment Survey.pdf [file mep-15-10861-s001.zip › B. Facilitator Guide.docx]

Appendix B

**Holistic Healthcare with Transgender Patients:** **Facilitator Guide**

**Learning Objectives**

By the end of this session students will be able to:

1. Describe the unique and overlapping professional roles and responsibilities of the providers on a healthcare team
2. Communicate effectively as an interprofessional healthcare team during a team huddle and a discharge planning simulation for a transgender patient
3. Develop shared ethics as an interprofessional team during a simulation experience
4. Critique interprofessional teamwork during a team huddle and simulated discharge planning meeting
5. Apply affirmative practice skills with a transgender patient during a simulated discharge planning meeting

**Facilitators Materials and Pre-Session Work**

1. Attend/watch the facilitator training
2. Read this faculty guide
3. Review the simulated emergency room video
4. Fill out the IP Core Competencies Adherence Handout for ED Video
5. Review the “Standardized Patient Case” document for the discharge planning activity
6. Review the “Team Huddle and Discharge Planning Behaviors” document
7. Review the reading materials sent to all students prior to the workshop

**Event Timeline** (Suggested time allowance for each step)

- **10 minutes** – Students check in for Workshop in
- **15 minutes** – Facilitators check in and meet SP and then all facilitators will walk up as a group to meet students
- **15 minutes** – Students will watch the simulation video of the patient in the ED and take notes
- **10 minutes** - Facilitators take assigned group to assigned seminar room – they will be seated all together by team and an announcement will be made for each team to go with their facilitator
- **15 minutes -** Facilitators will ask the students to discuss the video and relate what they observed to what they learned from the pre-event readings. (IPEC competencies, transgender affirmative practices, workmen’s comp, etc.)

Facilitator prompts to the students:

- - How did you observe the team uphold the four core IP competencies?
  - How did you observe the team violate the four core IP competencies?
  - What impact did the team’s behavior have on the patient?
  - What did you learn from the pre-workshop readings that informed your observations?
- **10 minutes -** Facilitators will then read the students the following prompt:

“It is now a couple of hours after this scene with Peter took place. Given Peter’s concern about his ankle, the social worker asked Dr. Thompson to have one of the hospital’s orthopedic surgeons take a look at the x-ray before discharge. The orthopedic surgeon reviewed the x-ray and made a note in the EMR that it is severly sprained but not broken. Good news is that he will not need surgery, but he will need a boot and crutches for 4 weeks. Peter will be able to get the boot/crutches before he leaves this evening, but he will still need to follow up with a local orthopedist in the next week for ongoing supervision of the ankle. The social worker requested a team meeting with Peter, to inform him about needing the boot and crutches, as well as to talk about next steps and possible referrals regarding his after-care, particularly related to his work situation, his living situation, or other aspects of his health such as his diabetes, before he is discharged from the hospital. As a team you will be meeting with Peter for about 20 minutes, so to prep for that meeting, you are now going to do a 10 minute team huddle where everyone should identify what questions you still have for the patient, what concerns of the patient you still need to address, and possible next steps for discharge including referrals. Your conversation should draw on what you saw in the video as well as information you learned in the pre-workshop readings (on differing health care team provider roles/responsibilities and needs/rights of transgender patients). You should also try to incorporate the following interprofessional practice behaviors. *[Facilitator reads the 7 interprofessional practice behaviors from the team huddle document]*. You have 10 minutes, which start now.”

- **15 minutes–** Facilitators then ask students to discuss their teamwork during the huddle and also provide feedback if needed. Facilitators can use the SP Discharge Planning Guide to offer suggestions for areas not generated/discussed by the students. Facilitators can also encourage incorporation of interprofessional practice behaviors from list.

Facilitators will then read the students the following prompt:

“As a team, reflect on your abilities to engage in interprofessional practice behaviors (facilitation redirect back to handout). What stands out to you as a team? One thing I observed was…(if needed). Take a look at the interprofessional practice behaviors related to the discharge planning meeting. As a team is there anything you want to pay close attention to or be mindful of as you go into the meeting with Peter?

When Peter comes in, be sure to introduce yourselves and your discipline, and someone needs to take the lead to tell Peter that his ankle is severely sprained and while he will not need surgery, he will need a boot/crutches for 4 weeks and needs to follow up with a local orthopedist.”

- **30 minutes** – Facilitators will need to make sure there is a chair at the front of the room for the patient and have the students sit close in a semicircle or circle depending on the room. Peter will then join the team and students should work as a team to ask their questions, share ideas, and talk about potential next steps that they had discussed in the team huddle, while seeking input from Peter about what he is most concerned about. Given the time constraint, they don’t have to solve everything – rather the focus is on the teamwork that they demonstrate and their ability to help Peter feel more comfortable leaving the hospital using effective team work and communication. Each student’s input is valuable. The last five minutes should be the standardized patient giving feedback to the student team. Was there any aspect that was uncomfortable or that the SP would have liked the students to word differently? What in particular did the students do well? After five minutes, facilitators should thank the SP for their time and participation and then let them know they can leave.
- **10 minutes –** Students will discuss their teamwork skills and then facilitators will give feedback to the student team based on behaviors on the interprofessional practice behaviors handout. Facilitators will then read the students the following prompt:

“As a team, reflect on the interprofessional practice behaviors on the handout... What stands out to you as a team? Is there something you feel as a team you improved upon from the huddle? One thing I observed was…(if needed). What do you think was your team’s greatest strength? Area for improvement if you could do it again or had a longer time with the patient?”

- **10 minutes –** Facilitators take students back to room for a large group debrief
- **20 minutes -** Students have a large group debrief and receive online survey
- **15 minutes -** Facilitators debrief the event and receive online survey

**___________________________________________________________________________**
