# Supplementary material for: Promoting Affirmative Transgender Health Care Practice Within Hospitals: An IPE Standardized Patient Simulation for Graduate Health Care Learners
Source: MedEdPORTAL. 2019 Dec 13;15:10861. doi: 10.15766/mep_2374-8265.10861 (PMC7010321; doi:10.15766/mep_2374-8265.10861)
Supplement: Supplementary file 1 — A. Logistical Requirements.docx B. Facilitator Guide.docx C. Standardized Patient Case Development Tool.docx D. IP Core Competencies Critique for ED Video.docx E. IP Behaviors for Team Huddle and Discharge Planning.docx F. ED Video.mp4 G. Guidelines for Student and Facilitator Debriefs.docx H. Posttest Assessment Survey.pdf [file mep-15-10861-s001.zip › C. Standardized Patient Case Development Tool.docx]

**Appendix C: Standardized Patient Case Development Tool**

Date: 2/1/2019

Primary Case Authors: Aaron W. Bernard, Gabbriel Ceccolini, Emily McCave

Secondary Case Authors: Rachel Feldman, Kim Hartmann, Fiore Salvador, Teresa Twomey, Rebecca Zucconi

Standardized Patient Educators: Aaron W. Bernard, Gabbriel Ceccolini

Name of Case: Transgender healthcare following workplace assault

Name of educational and or assessment activity:  Holistic Healthcare with Transgender Patients IPE Simulation

Patient Name: Peter Jacobs (Samantha Jacobs is assigned birth name)

Chief Complaint: Right ankle pain

Most likely Diagnosis and Differential with rationale from history and/or physical exam: Right ankle fracture

Challenge question:  Establish a care plan for a trauma patient after an assault through effective interprofessional collaboration while demonstrating respectful, gender-affirming practices.

Domains: Check all that apply

- Professionalism
- Communication and Interpersonal skills
- Medical History
- Physical exam
- Shared Decision Making
- Patient Education
- Clinical Reasoning
- Documentation
- Handoff
- Presentation
- Other: Interprofessional collaboration, holistic care for transgender patients

Type and level of learner: Interprofessional graduate learners from nursing, occupational therapy, physical therapy, social work, physician assistant, healthcare administration, and medical school degree programs.

Case Objectives:

By the end of this activity participants will be able to:

1. Describe the unique and overlapping professional roles and responsibilities of the providers on a healthcare team
2. Communicate effectively as an interprofessional healthcare team during a team huddle and a discharge planning simulation for a transgender patient
3. Develop shared ethics as an interprofessional team during a simulation experience
4. Critique interprofessional teamwork during a team huddle and simulated discharge planning meeting
5. Apply affirmative practice skills with a transgender patient during a simulated discharge planning meeting

| SETTING: | Emergency Department |
| --- | --- |
| PATIENT PROFILE: You are a transgender man (assigned female at birth) who presents with a right ankle injury after an altercation.  The students will watch a video of the initial ED encounter.  You will then meet with the students in a small group. They will ask further questions not covered in the video and then plan with you for your safe discharge from the Emergency Department. The goal is for the students to consider your needs (medical, occupational, physical, and social), treat you in a respectful and affirmative manner, and ensure you have follow up care after you leave. | |
| Age range | All may be used |
| Religious/spiritual background | All may be used |
| Sex | Assigned female at birth |
| Gender Identity | Transgender male |
| Pronouns | He/His/Him |
| Sexual Orientation | Both male and female partners in the past |
| Gender Expression | Man |
| Race/ethnicity | All may be used |
| Physical Description | All may be used |
| Physical Limitations | Ace bandage on right lower leg/ankle and shoe removed from that foot. |
| Patient Appearance | SP should refrain from wearing make-up and dress in clothing that would lead them to be perceived as male (button down shirt, baggy t-shirt, jeans, etc). If they have long hair, they should have hair back in ponytail if possible. A hat is another option. |
| Moulage | None |
| Affect | Nervous/anxious about talking to health care providers (particularly after the initial ED experience), still a bit emotional (frustrated, fearful) consequent to the assaultive behavior of their co-worker. Poor eye contact. |
| Family Group | Parents are in Midwest (where you grew up) but has been on east coast for many years. Rarely speaks to or sees his parents. Does not have any close friends mostly due to limited transportation and changing work schedule. He has seen a counselor in the past a couple of years ago. He has gone to a support group for individuals who are transgender, but only goes sporadically due to his work schedule. |
| Education | High School |
| Level of health literacy | Proficient |
| Home | Lives alone in an apartment, 2^nd^ floor, sometimes elevator is out of operation. |
| Financial Situation | Finances are poor. Difficulty paying bills. No car; uses bus to get to work. |
| Insurance Status | State health insurance. |
| Habits | Social drinker (2-3 drinks on a weekend night). No recreational drugs or tobacco. A few cups of coffee in the morning. |
| Activities | Enjoys watching TV (cooking shows, sci-fi shows) |
| Typical Day | Works in a fast food restaurant. |

| CASE INFORMATION | |
| --- | --- |
| Chief Concern: | “Right Ankle Pain”  (Learners will watch the initial ED encounter on a video.) |
| Additional Concerns: | “I need to get back to work to make money and pay my bills. I don’t see how I can do it if I’m on crutches. I don’t know how I’m going to keep on top of everything. And I don’t know how I am getting home on crutches.” |
| THE PATIENT STORY: | Learners will review the video of the initial ED encounter to have a sense of what was already discussed prior to this discharge planning session. In brief, you had a variety of difficult interactions with the ED staff and have been told you are being discharged from the ED. You expressed concern about your readiness to be discharged to the social worker and now a group of professionals (PT, OT, MD, RN, SW) are going to talk to you, get more information, and figure out a safe discharge plan. |
| HISTORY OF PRESENT ILLNESS: You are a transgender man (assigned female at birth) in transition who presents with a right ankle injury after being physically assaulted by your co-worker in the bathroom at your workplace.  You have been socially transitioning for 1 year – meaning you have changed your name and pronouns for social interactions and are making others aware, when needed, about your gender identity. You also have been expressing your gender with more masculine clothing. You have not yet started any legal transition yet, such as legally changing your name, which is why your insurance card still has your assigned name on it. You have started hormone therapy (testosterone) in order to create more masculine characteristics such as a deeper voice and facial hair. You are considering additional medical transition including getting “Top Surgery” (gender-affirming surgery - specifically a mastectomy – though it is a different medical technique that is used for trans men) and are saving up the $8,000 that you believe it will cost to pay for it. (You are unaware that Medicaid will actually pay for it in your state). You see a specialist (endocrinologist) for your transitional care as well as for your diabetes management. You have not yet found a primary care doctor who you feel comfortable with for routine preventative screenings and non-urgent health needs. You have not seen a gynecologist in 5 years. You have a podiatrist for your foot ulcer but you don’t like going because of her staff are uncomfortable around you.    You have been working at McDonalds for a few months. One co-worker has always been somewhat standoffish with you since the time you started. More recently in the last two weeks he has told you in passing he does not think you should be using the male bathroom. This has created an uncomfortable work environment but the comments have been limited and you did not report them to your supervisor.  Earlier today, you went to the bathroom on break and this co-worker followed you in. Before you could use enter a stall he said you shouldn’t be in here. He was loud, aggressive, and intimidating in his tone. You told him to get out and leave you alone. He then proceeded to call you a pervert and said if you didn’t leave, he would make you. You tried to move to go into the stall but then he grabbed you and tried to force you physically out of the bathroom. In the physical struggled you got pushed down. You felt your ankle twist and as you tried to get up you found you couldn’t put any weight on it. You yelled for your manager, who called 911, and EMS brought you to the Emergency Department. You did not get to speak with the manager about what happened and you didn’t see the co-worker as you left.  Your pain in your right ankle is minimal because you have bad “neuropathy” from your diabetes and don’t have much feeling to begin. EMS said they are not sure if it is broken or a severe sprain. Your pain is 3/10, non-radiating, sharp, worse with movement, better with rest. There are no associated injuries.  You are nervous/anxious about going to the ED and talking to the hospital staff and providers. You have had bad experiences in the past with health providers and their staff. You identify as a trans male and you want to be called Peter; you use he/him/his pronouns. You have had health care providers call you Samantha and use the pronoun “her” when talking to/about you. This upsets you. You have experienced other health care providers or their staff who seem awkward around you and sometimes judgmental. For these reasons, you are anxious/nervous. Also, you have heard from members of the trans support group which you attend that some of them have been refused emergency medical care because they were trans.  You would also self-describe yourself as emotional, upset, and scared as it relates to the altercation with your co-worker. You feel you are just trying to do your job and live your life and people like this co-worker go out of their way to make your life harder and to try to bully you into being someone you’re not. | |
| Review of Systems: | None significant |
| Past Medical History: | Diabetes: You have type 1/insulin requiring/childhood onset diabetes. You developed it at the age of 5. You use insulin and manage it as best you can, given your limited access to healthy foods (costly, you get a discount at McDonald’s, and not easy to find in your neighborhood) and daily stress.   - Your blood sugar at the moment is 180 (EMS checked). It is normally 180-300. Your Hemoglobin A1C (level checked by a doctor) is typically about 10.0 (<7.0 for diabetics preferred) - Diabetic neuropathy You have little sensation in your injured foot due to neuropathy. It causes you some pain but more numbness. This is why your sprained ankle doesn’t hurt much at the moment. - Diabetic ulcer You have an ulcer on the base of your right foot. This is related to the diabetes as well. Currently it is being treated by putting more padding under it and in your shoes. - Your endocrinologist treats your diabetes and prescribes your insulin. You also see this provider for your hormone treatment. You feel comfortable with this provider.   Eye doctor (for diabetes) you are supposed to go to yearly but you don’t.  Foot doctor (for diabetes) you only started to go to when the ulcer developed but you don’t go every month as you were told. |
| Medication allergies | None |
| Environmental allergies | None |
| Illnesses | As above |
| Vaccinations | Not up to date with flu vaccine |
| Surgeries | None |
| Accidents/ injuries/ trauma | None |
| Hospitalizations | None |
| Sexual History | Both male and female partners in the past. No current partner, not sexually active in the past year. It’s been hard to meet people who are accepting of you during your transition and you don’t really have the time or money to go out on dates. You have also heard some horror stories about trans folks getting beat up on dates after the partner finds out they are trans.  Uses condoms, no history of STI. |
| Ob/Gyn History | Menarche 12, Menopause 58 (if applicable).  No pregnancies.  Last menstrual period was before starting hormone therapy (if applicable).  Last mammogram 5 years ago (if over 40).  Last pap smear 5 years ago. |
| Medications | - Testosterone IM 1x/week for medical transition - Lantus 30 units SC qhs, for diabetes - Novolog 8 units SC TID, sliding scale with each meal, for diabetes |
| Immunizations | - Tetanus up to date - No Flu in several years - No Hepatitis - No Pneumovax - No HPV |
| Tobacco products | None |
| Alcohol | 2-3 drinks weekly |
| Drugs | None |
| Diet | Frequent fast food |
| Exercise | None |
| Family History | - Mother alive (25 years older), Type 2 Diabetes - Father alive (25 years older), Hypertension - No siblings - No known details about extended family |
| PHYSICAL EXAM: | Not performed in this simulation |
| **DISCHARGE PLANNING SESSION:** | - The SP will meet with a group of students and one/two facilitator(s). - They will first ask the SP a series of questions to develop a deeper understanding of the situation. - They then will ask the SP about their feelings toward a series of treatment and intervention options. - Below are a number of questions that students may ask. |

| **Areas Expected to be Addressed During Discharge Planning Meeting** | **General Answer (does not need to follow the script)** |
| --- | --- |
| *Hi Peter – I know you had some concerns about leaving the hospital before you felt ready. We wanted to meet with you to discuss a discharge plan that you’re comfortable with. We want you to know that we had an orthopedic doctor look at your X-ray and she said you do not need surgery, but you will need a boot and crutches for 6-8 weeks, which we can give you here at the hospital before you leave tonight. You will also need to see a local orthopedic doctor in the next week for follow up care. We want to ensure you have all the pieces put into place to make the discharge transition a smooth one. I’m curious what is most important to you as you think about leaving the hospital?* | “I need to get back to work to make money and pay my bills. I don’t see how I can do it if I’m on crutches. I don’t know how I’m going to keep on top of everything. And I don’t know how I am getting home on crutches.” |
| *I’m wondering if you’d like to talk with a professional about your situation at work and the incident to explore your options?*  *You could talk to them about filing for workman’s compensation; it would allow you to still get paid but not have to go to work while your ankle is healing. It would also mean you wouldn’t be able to sue McDonald’s for negligence.* | “Yeah, I don’t think I can work on crutches – I’d like to maybe get workman’s comp as long as it doesn’t cause any trouble at work. I need this job.” |
| *You mentioned worrying about going back to work with your co-worker being there – did you want to speak with someone about your legal rights to have a safe workplace?*  *You might consider going to a legal aid clinic for free legal care in case you want to pursue legal action against his co-worker* | “I don’t know what they can do, but I just want to go in to the bathroom in peace. I don’t want any trouble.” |
| *Describe your roles and tasks at work; What are your job duties? How long is your typical work day? What percentage of the day do you stand/sit?* | “Sometimes I work from 6am -2:30pm, sometimes 2:30 -11pm – every once in awhile I have to do a night shift - I do a bit of everything – clean up/cashier, fryer. I stand pretty much the whole day.” |
| *Can you tell me about your living situation?* | “I live in a one-bedroom apartment – it’s on the 2^nd^ floor of an apartment building. There’s an elevator but I usually don’t take it because sometimes it doesn’t work and I’ve gotten stuck on it. My landlord doesn’t seem to care – he’s hard to get a hold of.” |
| *Describe your apartment, meal preparation needs, and personal hygiene routines that may be impacted by the injury (or crutch use). What kind of floors/carpeting/rugs do you have in your apartment? Do you have a walk-in shower or a tub or a shower in the tub? Do you have any grab bars?* | “Mostly I go to the little corner store and some fast food places just a couple of block from me since it’s easier then going all the way to the grocery store a few miles away. I have carpet in the whole apartment – and a regular tub and shower. No grab bars.” |
| *Can anyone help you getting things like groceries into the apartment? What other things do you anticipate having difficulty doing while you are walking on crutches?* | “There’s a lady down the hall who is pretty nice – but she isn’t always around.  I’m worried about working mostly and getting up the stairs to my apartment and walking to the bus stop.” |
| *Would you be open to having an OT follow up with you and come to your place to help you figure out how to best carry out your daily activities, like meal prep?*  *Evaluation on-site of the work site as part of either worker compensation or task re-assignment or modification. Include in the work evaluation additional methods to increase social participation and healthy living.*  *Request a referral for work assessment for other work options-paid for via worker’s compensation OR job accommodation network.*  *Consider a referral to a nutritionist through the primary health care provider.*  *Provide instruction on using the bathroom – shower/tub, toilet*  *Discuss risks for falls inside the apartment e.g. remove scatter rugs, not using towel rack to get up from toilet*  *Education about how to dress and what kinds of clothing would be easiest to put on/take off.* | “Yeah, that would be helpful, I think.” |
| *Before you leave – we want to have you meet once more with PT here. You’ll go over crutch training on levels and stairs. They will discuss option of knee scooter and do sensation testing and balance testing as this may impact safety with crutches. Also, examine wound and discuss the need for greater oversight with this due to the risks of wounds developing into serious problems in people with diabetes. Education regarding donning and doffing brace, skin inspection, and gait training on level and stairs.* | “Okay, that sounds good. I’ve never used crutches before.” |
| *Can you tell me about your access to transportation – do you have a car?* | “No, I just take the bus where I need to go, or walk it. I have to walk about a half mile to get to the bus stop near my place. I can get where I need to go from there – the little corner store and some fast food places just a couple of block from me so that’s easier then going all the way to the grocery store a few miles away.” |
| *Before you leave, we want to make sure you know how to use ice/elevation and over the counter medications to make yourself more comfortable at home.* | “Okay, that’d be good to know. I usually just take ibuprofen. I don’t have any ice packets or anything at my place.” |
| *I know you mentioned that you have your podiatrist for the foot ulcer as well as your specialist for your testosterone and diabetes management – do you have any upcoming appointments that you need help getting to?*  *Would like some other recommendations for doctors who we know would make sure you are treated better?* | “Yeah, I have an appointment coming up for my podiatrist but I hate going there. I think I need a switch at this point. I do worry about my foot, but I want to go someplace where people treat me right.” |
| *Would you like some information on how to manage your own foot care or your diabetes while you recover from this injury?* | “I know I just need to eat better – but it’s not easy. It’d be nice if I could get a ride to the grocery store once in awhile and get some healthier foods. I probably need someone to show me again how to take care of the sore on my foot.” |
| *Would you like to reach out to a support person from your trans group for extra support? Maybe regarding strategies for dealing with possible future issues related to a safe and comfortable work place?* | “I have the phone numbers of a couple of them; I can give them a call I guess. They at least get what I’m going through.” |
| *You mentioned you went to a trans support group – do you have an individual counselor who you could get some extra support from?* | “Yeah maybe – I was seeing someone a couple of years ago but it’s a hike getting there so I’d have to get a ride. It was one of the reasons I stopped going – just a pain to get there without a car.” |
| *Have you ever been attacked or hurt someplace else when trying to use the men’s bathroom?* | “No, thank god. But I’ve gotten some really nasty comments and glares at restaurants and at the movies. I usually try to wear a baseball cap and keep my head down, you know? You never know who is out there and what they might do. It’s like I’m always looking over my shoulder. I read about all these stories on the news – trans people are getting killed over this bathroom stuff. It’s crazy.” |
| *You mentioned that you are on state insurance and that you were saving up for top surgery – I was wondering if you knew that the state insurance will actually cover sex reassignment surgery?* | “What? Really? I had no idea – I had heard from one of the guys from group that he had to pay like 8 grand out of pocket – but I guess he did have private insurance! That’s a huge relief!” |
| *I’m wondering if you aware of how your diabetes management may impact your healing from any transition surgery you have?*  *Perhaps redirection back to the endocrinologist, or some discussion if the patient is aware of recommended blood sugar/A1c targets and unintended consequences of uncontrolled T1D?*  *May require insulin adjustment, ensuring adherence to meds and TLC, proper pairing of meal-time insulin with carbohydrate intake, etc…* | “I am not really sure. What do I need to know?” |
| *Also, with state insurance, we can get medical transportation arranged at no cost -so that you can get to your doctor’s appointments. We could also look at what options for transportation we can arrange while you are on workman’s comp.* | “That would be great actually – I think I just need to know how it works – like how I set it up.” |
| *How do feel now about leaving the hospital?* | “I’m still a little nervous but I feel like at least I have some things in place and I’m going to be okay.” |
