# Supplementary material for: Promoting Affirmative Transgender Health Care Practice Within Hospitals: An IPE Standardized Patient Simulation for Graduate Health Care Learners
Source: MedEdPORTAL. 2019 Dec 13;15:10861. doi: 10.15766/mep_2374-8265.10861 (PMC7010321; doi:10.15766/mep_2374-8265.10861)
Supplement: Supplementary file 1 — A. Logistical Requirements.docx B. Facilitator Guide.docx C. Standardized Patient Case Development Tool.docx D. IP Core Competencies Critique for ED Video.docx E. IP Behaviors for Team Huddle and Discharge Planning.docx F. ED Video.mp4 G. Guidelines for Student and Facilitator Debriefs.docx H. Posttest Assessment Survey.pdf [file mep-15-10861-s001.zip › D. IP Core Competencies Critique for ED Video.docx]

Appendix D (Table Created by Authors)

**Holistic Healthcare with Transgender Patients –IP Core Competencies Observed During ED Video (Handout)**

| *Interprofessional Core Competencies for Collaborative Practice (IPEC, 2016, p. 10)* | *Team Behaviors Demonstrating Mastery of this Competency:* | *Team Behaviors that Violate this Competency:* |
| --- | --- | --- |
| 1. Work with individuals of other professions to maintain a climate of mutual respect and shared values **(Values and Ethics)**. |  |  |
| 2. Use the knowledge of one’s own role and those of other professions to appropriately assess and address the health care needs of patients and to promote and advance the health of populations. **(Roles/Responsibilities)** |  |  |
| 3. Communicate with patients, families, communities, and professionals in health and other fields in a responsive and responsible manner that supports a team approach to the promotion and maintenance of health and the prevention and treatment of disease. **(Interprofessional Communication)** |  |  |
| 4. Apply relationship-building values and the principles of team dynamics to perform effectively in different team roles to plan, deliver, and evaluate patient/population centered care and population health programs and policies that are safe, timely, efficient, effective, and equitable. **(Teams and Teamwork)** |  |  |

Interprofessional Education Collaborative. Core competencies for interprofessional collaborative practice: 2016 update. Washington, DC: Interprofessional Education Collaborative. Published 2016. Accessed January 25, 2019.

[**https://nebula.wsimg.com/2f68a39520b03336b41038c370497473?AccessKeyId=DC06780E69ED19E2B3A5&disposition=0&alloworigin=1**](https://nebula.wsimg.com/2f68a39520b03336b41038c370497473?AccessKeyId=DC06780E69ED19E2B3A5&disposition=0&alloworigin=1)
