# Supplementary material for: Promoting Affirmative Transgender Health Care Practice Within Hospitals: An IPE Standardized Patient Simulation for Graduate Health Care Learners
Source: MedEdPORTAL. 2019 Dec 13;15:10861. doi: 10.15766/mep_2374-8265.10861 (PMC7010321; doi:10.15766/mep_2374-8265.10861)
Supplement: Supplementary file 1 — A. Logistical Requirements.docx B. Facilitator Guide.docx C. Standardized Patient Case Development Tool.docx D. IP Core Competencies Critique for ED Video.docx E. IP Behaviors for Team Huddle and Discharge Planning.docx F. ED Video.mp4 G. Guidelines for Student and Facilitator Debriefs.docx H. Posttest Assessment Survey.pdf [file mep-15-10861-s001.zip › E. IP Behaviors for Team Huddle and Discharge Planning.docx]

Appendix E (Created by Authors)

**Holistic Healthcare with Transgender Patients – IP Behaviors for Team Huddle and Discharge Planning Meeting**

As a facilitator, please remind students that they should be considering the following behaviors before they start their team huddle and then later the discharge planning meeting.

| **INTERPROFESSIONAL PRACTICE BEHAVIORS** |
| --- |
| ***During the team huddle, your team should….*** |
| use the appropriate pronoun/name when talking about the patient |
| prioritize the questions/concerns brought up by the patient |
| be non-judgmental of the patient’s situation |
| demonstrate knowledge of needs/rights/experiences of transgender individuals |
| seek input from each team member |
| respectfully consider all team members’ perspectives |
| demonstrate shared leadership in determining questions/plan for discharge meeting |
|  |
| ***During the discharge planning meeting, as a team you should…*** |
| use the appropriate pronoun/name when talking about/with the patient |
| demonstrate knowledge of needs/rights/experiences of transgender individuals |
| clearly describe different roles of providers to patient |
| seek input from patient about needs/concerns |
| prioritize the needs, feelings, and care goals of the patient |
| recognize multiple health disciplines needed to promote overall patient well-being |
| demonstrate shared leadership among all team members |
