# Supplementary material for: Promoting Affirmative Transgender Health Care Practice Within Hospitals: An IPE Standardized Patient Simulation for Graduate Health Care Learners
Source: MedEdPORTAL. 2019 Dec 13;15:10861. doi: 10.15766/mep_2374-8265.10861 (PMC7010321; doi:10.15766/mep_2374-8265.10861)
Supplement: Supplementary file 1 — A. Logistical Requirements.docx B. Facilitator Guide.docx C. Standardized Patient Case Development Tool.docx D. IP Core Competencies Critique for ED Video.docx E. IP Behaviors for Team Huddle and Discharge Planning.docx F. ED Video.mp4 G. Guidelines for Student and Facilitator Debriefs.docx H. Posttest Assessment Survey.pdf [file mep-15-10861-s001.zip › G. Guidelines for Student and Facilitator Debriefs.docx]

Appendix G

**Guidelines for Student and Facilitator Debriefs**

Large Student Debrief

- Start with an open-ended question – what was most impactful during your experience today?
- Other questions to ask during debrief
  - What was most challenging during your team huddle experience or discharge planning meeting?
  - In thinking about your team, what is something your team did that stood out as most helpful? (Tie it to the four core competencies if possible)
  - How will this impact your future practice with patients, both those who are transgender and those who are not?

Facilitator Debrief

- Start with an open-ended question – what stood out to you as observed your student teams during the simulation experience?
- Other questions to ask during debrief
  - What were any challenges that you experienced as a facilitator during either the team huddle experience or discharge planning meeting?
  - Where do you think the students struggled the most during the experience? Where did they excel?
  - What could we do better next time to reach our learning objectives?
