# Supplementary material for: Promoting Affirmative Transgender Health Care Practice Within Hospitals: An IPE Standardized Patient Simulation for Graduate Health Care Learners
Source: MedEdPORTAL. 2019 Dec 13;15:10861. doi: 10.15766/mep_2374-8265.10861 (PMC7010321; doi:10.15766/mep_2374-8265.10861)
Supplement: Supplementary file 1 — A. Logistical Requirements.docx B. Facilitator Guide.docx C. Standardized Patient Case Development Tool.docx D. IP Core Competencies Critique for ED Video.docx E. IP Behaviors for Team Huddle and Discharge Planning.docx F. ED Video.mp4 G. Guidelines for Student and Facilitator Debriefs.docx H. Posttest Assessment Survey.pdf [file mep-15-10861-s001.zip › H. Posttest Assessment Survey.pdf]

Appendix H (Created by Authors)

**Holistic Healthcare with Transgender Patients – Post-Test Assessment Surveys for Students and Facilitators**

**Student:**

1. My participation in this workshop was: Voluntary

2. My discipline of study is: Social Work

3. This workshop increased my awareness of the specific healthcare needs of transgender patients and clients. 5 = Strongly Agree

4. This workshop increased my awareness of the importance of asking about the gender identity of my patients. 5 = Strongly Agree

5. This workshop increased my comfort in communicating with transgender patients and clients in an appropriately sensitive manner. 5 = Strongly Agree

6. Did you complete the pre-session reading assignment? No

   a. If yes, please answer the following: The pre-session readings provided useful information about
      i. LGBT terminology 5 = Strongly Agree
      ii. LGBT healthcare practices 5 = Strongly Agree
      iii. LGBT legal rights 5 = Strongly Agree
      iv. IPE core competencies 5 = Strongly Agree
      v. health care team roles 5 = Strongly Agree

   b. The length of the assigned reading was: Just right

7. Please rate each of the following aspects of the workshop in terms of their value in educating you about transgender patient issues.
   a. ED Video 1 = Not Useful
   b. Team Debrief on Video as it Relates to IP Core Competencies 1 = Not Useful
   c. Team Huddle 1 = Not Useful
   d. Discharge Planning Meeting with Patient 1 = Not Useful
   e. Large Student Debrief 1 = Not Useful

**8. Please provide any other feedback you would like to share with us: (open ended text box)**

**Facilitator:**

1. My discipline is: Social Work

2. I have experience working with transgender patients within my discipline. 5 = Strongly Agree

3. This workshop provided opportunities to appreciate the unique needs of transgender patients across many disciplines. 5 = Strongly Agree

4. This workshop provided strategies for being inclusive and affirmative with transgender patients.
   5 = Strongly Agree

5. This workshop increased my awareness of the following:
   a. transgender patient health needs 5 = Strongly Agree

   b. IPE core competencies 5 = Strongly Agree

   c. health care team roles 5 = Strongly Agree

6. This workshop contributed to the University's efforts to promote Inclusion and Diversity. 5 = Strongly Agree

**7. Please provide any other feedback you would like to share with us: (open ended text box)**
